# Low-Odor High-Density Fiberboard Enabled by Supramolecular Interactions in Wood Fibers

**DOI:** 10.3390/polym18020297

**Published:** 2026-01-22

**Authors:** Xia Yu, Zongying Fu, Bo Liu, Xiaoxuan Guo, Yun Lu, Lihong Yao

**Affiliations:** 1 College of Material Science and Art Design, Inner Mongolia Agricultural University, Hohhot 010018, China; xyu02032023@163.com; 2 Key Laboratory of Wood Science and Technology of National Forestry and Grassland Administration, Research Institute of Wood Industry, Chinese Academy of Forestry, Beijing 100091, China; zyfu@caf.ac.cn (Z.F.); liubo@caf.ac.cn (B.L.); guoxx901@163.com (X.G.)

**Keywords:** oxidative treatment, tannic acid, supramolecular interactions, low-odor, high-density fiberboard

## Abstract

The development of sustainable wood-based composites has driven increasing interest in formaldehyde-free, low-odor, and recyclable bonding systems. However, achieving high mechanical performance and dimensional stability in high-density fiberboards (HDFs) without synthetic adhesives remains a challenge. Here, we report a two-step strategy combining oxidative pretreatment of wood fibers with supramolecular assembly of tannic acid (TA) and sodium ions (Na^+^) to fabricate low-odor, recyclable HDF. Oxidation generated abundant carboxyl groups on the fiber surface, enabling strong coordination and hydrogen-bonding interactions between TA and Na^+^, which constructed robust inter-fiber supramolecular networks without formaldehyde-based adhesives. The resulting HDF exhibited excellent mechanical properties, with an internal bond strength of 3.1 MPa, a modulus of rupture of 49 MPa, and 24 h water thickness swelling of only 12%. Odor and VOC analysis revealed only trace benzene, demonstrating markedly low odor. Furthermore, the reversible nature of Na^+^-TA interactions allowed efficient fiber separation and recyclability under mild aqueous conditions. This oxidation-assisted supramolecular approach provides a sustainable route for producing high-performance, low-odor, and recyclable fiberboards, offering a viable alternative to conventional polymer-bonded wood composites.

## 1. Introduction

Wood-based fiberboards, particularly high-density fiberboard (HDF), are widely employed in furniture, interior decoration, and construction due to their excellent mechanical properties, dimensional stability, and cost-effectiveness [1]. Conventional HDF fabrication relies heavily on synthetic thermosetting resins, such as urea–formaldehyde (UF) [2,3] and phenol–formaldehyde (PF) adhesives [4,5]. While these adhesives provide reliable bonding performance, they inevitably release volatile organic compounds (VOCs) and associated odor emissions, which have raised significant concerns regarding indoor air quality, human health, and environmental sustainability [6,7,8]. Consequently, developing formaldehydefree, low-odor, and environmentally friendly bonding strategies has become a major focus in the advancement of wood—based composites [9,10,11].

In recent years, various polymer-free and bio-based approaches have been explored to replace synthetic adhesives [12,13,14], including lignin- and protein-based binders [15,16,17,18], as well as physical self-bonding techniques [19,20]. Among these strategies, supramolecular interactions [21,22]-such as hydrogen bonding, metal-ligand coordination [23,24], and ionic interactions [25,26] have attracted particular attention due to their reversibility [27], adaptability, and potential recyclability [28,29]. Polyphenolic compounds [30], especially tannic acid (TA) [31], are promising candidates for supramolecular bonding owing to their abundant phenolic hydroxyl groups and strong affinity toward polysaccharides and metal ions [32,33]. However, when TA is directly applied to native wood fibers—i.e., oven-dried fibers that have not undergone any chemical modification or pre-treatment-the bonding efficiency is often limited due to insufficient interfacial interactions and restricted accessibility of reactive sites, resulting in reduced mechanical performance and diminished dimensional stability [34,35]. Oxidative pretreatment of wood fibers has emerged as an effective strategy to overcome these limitations by introducing additional reactive functional groups, such as carboxyl groups, onto the fiber surface [29,36]. These functional groups significantly enhance interfacial interactions and facilitate the formation of supramolecular networks [37]. Furthermore, alkali metal ions, such as sodium ions (Na^+^), can act as coordination centers to bridge TA molecules and oxidized fiber surfaces, reinforcing inter-fiber bonding through synergistic hydrogen bonding and ionic coordination. Despite these advantages, the integration of oxidation-assisted supramolecular assembly in HDF fabrication—particularly for achieving high mechanical strength, low odor emissions, and recyclability—remains underexplored [38,39].

**Figure 1:**
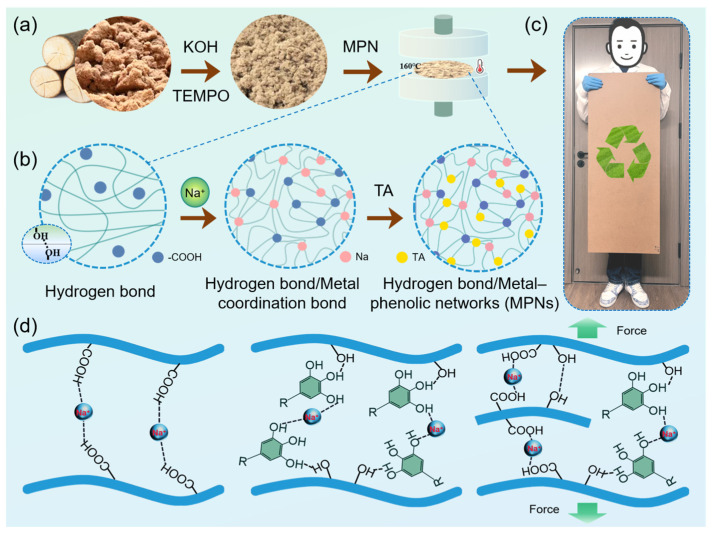
Schematic diagram of OWFB-Na-TA preparation and its mechanism. (**a**) Fabrication process of OWFB–Na–TA by pretreated hot pressing; (**b**) Schematic illustration of coordination between sodium ions and tannic acid during OWFB–Na–TA fabrication; (**c**) Photograph of the fabricated OWFB–Na–TA; (**d**) Mechanistic schematic of the pretreated hot pressing process for OWFB–Na–TA.

In this study, an oxidation-assisted supramolecular strategy is proposed for the fabrication of formaldehyde-free, low-odor, high-density fiberboards. In this approach, wood fibers are first subjected to oxidative pretreatment to enhance surface reactivity, followed by sodium ion treatment and subsequent tannic acid-mediated supramolecular assembly to construct inter-fiber bonding networks (Figure 1). The objective of this work is to elucidate how oxidation and supramolecular interactions influence fiber bonding behavior, structural integrity, and odor emission, providing a feasible approach for the production of green, binder-free wood-based products.

## 2. Materials and Methods

### 2.1. Materials

Poplar wood (*Populus* spp.) fibers were provided by Lingshou County Yigao Mineral Products Processing Plant Co., Ltd. (Shijiazhuang, Hebei, China). Eucalyptus wood (*Eucalyptus* spp.), Chinese fir wood (*Cunninghamia lanceolata* (Lamb.) Hook.), pine wood (*Pinus* spp.), and mixed hardwood fibers were provided by Taizhou Daziran Home Co., Ltd., (Taizhou, Jiangsu, China) in collaboration with Fenglin Group. All wood fibers were obtained as industrial fibers derived from fiberboard manufacturing processes. Potassium hydroxide (KOH, 95%), sodium chlorite (NaClO_2_, 80%), 2,2,6,6-tetramethylpiperidin-1-oxyl free radical (TEMPO, 98%), sodium hypochlorite (NaClO, active chlorine 13%~16%), anhydrous ethanol (CH_3_CH_2_OH, 99.5%), tannic acid (C_76_H_52_O_46_, 98%) and sodium chloride (AR, NaCl) were all purchased from Shanghai Macklin Biochemical Co., Ltd. (Shanghai, China). Phosphate buffer (pH = 6.86) was ordered from Sinopharm Chemical Reagent Co., Ltd. (Shanghai, China).

### 2.2. Oxidative Treatment of Wood Fibers

First, 20 g of oven-dried eucalyptus wood fibers were immersed in 500 mL of 2 wt% potassium hydroxide (KOH) aqueous solution and treated at 90 °C for 2 h. After hemicelluloses removal, the fibers were repeatedly washed with deionized water to remove residual chemicals. Subsequently, the delignified fibers were suspended in 220 mL of phosphate-buffered solution (pH 6.8), containing 0.032 g of 2,2,6,6-tetramethylpiperidine-1-oxyl (TEMPO), 2.825 g of sodium chlorite (NaClO_2_), and 0.64 mL of sodium hypochlorite (NaClO). The reaction was carried out in a beaker at 60 °C for 2, 6, 12, or 24 h. After the reaction, the fibers were washed with ethanol and deionized water, and the excess water was removed by squeezing. For the fabrication of larger-scale samples, all materials were proportionally increased while maintaining the same composition ratios as in the original formulation. Depending on the size of the hot press, boards of different dimensions can be fabricated, ranging from 300 × 300 mm to 1000 × 500 mm.

The fiberboards prepared from five different woody species without any chemical treatment were not subjected to the treatment steps and were directly included in Section 2.3 for discussion.

### 2.3. Preparation of Oxidized Wood Fiberboard (OWFB)-Na-TA

The mass percentages of sodium chloride (NaCl) and tannic acid (TA) relative to dry wood fibers are 1.64 wt% each. An appropriate amount of water was added to fully dissolve the reagents for spraying. The solution was sprayed onto the fibers while simultaneously stirring with a mechanical mixer to ensure uniform distribution. After all solutions were applied, the fibers were dried in an oven until reaching a moisture content of 21% (this value was determined from multiple preliminary experiments; the detailed process is proprietary). The treated fibers were then placed into a molding die and hot-pressed using a CREE-6014H-30 hot press (Beijing, China) at various temperatures (130 °C, 140 °C, 150 °C, 160 °C, 170 °C) under a pressure of 8 MPa for 10 min to produce OWFB-Na-TA. The board thickness was controlled at 3 ± 0.05 mm. The resulting board density ranged from 1.0 to 1.1 g·cm^−3^. To ensure comparability of mechanical and physical properties, boards prepared from different treatments or different types of wood fibers were carefully controlled such that their densities differed by no more than 0.1 g·cm^−3^. This density control allows the performance differences to be attributed primarily to the treatment and fiber type rather than variations in board density. Minor variations in fiber amount may occur due to differences in fiber size and species moisture content.

### 2.4. Microstructure and Chemical Properties Analysis

Scanning electron microscope (SEM) (GeminiSEM300, ZEISS, Oberkochen, Germany) and energy-dispersive X-ray (EDX) analysis were utilized to characterize the microstructure and elemental distribution of the samples. The samples were cut into small pieces (5 × 5 mm) using a mini table saw, and the testing surfaces were polished with a sliding microtome. The images of the sample morphology and elemental distribution were captured under a vacuum operation mode at 3.0 kV. Prior to SEM scanning, it is necessary to perform pre-sprayed with gold/palladium (Au/Pd) on the surface of the sample.

Fourier transform infrared spectroscopy (FTIR) was performed on a Thermo Scientific Nicolet iS20 spectrometer (Thermo-Fisher Scientific, Waltham, MA, USA) to determine the functional groups of wood samples. Then collect the infrared spectrum of the sample with a resolution of 4 cm^−1^, scan 32 times, and test the wavenumber range of 400–4000 cm^−1^. The relative contents and chemical valency of elements were determined by a Thermo Scientific K-Alpha type X-ray photoelectron spectroscopy (XPS) (Thermo-Fisher Scientific, Waltham, MA, USA). The XPS spectra were collected at 0–1200 eV and calibrated based on surface contamination C1s (284.8 eV) as the standard.

### 2.5. Moisture Content Determination

The moisture content was measured directly using a LICHEN/Lichen moisture analyzer (Model DHS-20A, Shanghai, China).

### 2.6. Characterization of Density

The equipment used is an electronic balance manufactured by Kunshan Youkeweit Electronic Technology Co., Ltd., (Kunshan, Jiangsu, China) model CN-LQC20002. The mass of the fiberboard is measured using the balance. The sample density is then calculated based on the mass and volume of the fiberboard. The calculation formula is shown in Equation (1).

(1) $$\rho =\frac{M}{V_{0}},$$

where ρ is the fiberboard density in g/cm^3^, *M* is the mass of the fiberboard in g, and *V*_0_ is the volume of the fiberboard in cm^3^.

### 2.7. Characterization of Mechanical Properties

Internal bonding strength and bending strength were tested using a KXWW-01C mechanics testing machine (Chengde Kebiao Testing Instrument Manufacturing Co., Ltd. Chengde, Hebei, China). According to GB/T 17657-2022 “Test Methods for Physical and Chemical Properties of Artificial Boards and Surface Decorated Artificial Boards” [40], the bending strength of the sample was tested using the three-point bending method. The sample size was 150 × 50 mm (length × width), with a gauge length of 50 mm and a loading speed of 2 mm/min. Each group was tested with 5 samples, and the average value was taken. The sample size for testing internal bonding (IB) strength was 50 × 50 mm, with a loading speed of 2 mm/min. Five samples were tested in each group, and the average value was taken.

Table saw: MicroJiang W1 table saw (Shenzhen Jiangong Trading Co., Ltd., Shenzhen, China).

Band saw: MBS 240/E mini band saw (PROXXON, Föhren, Germany).

### 2.8. Statistical Analysis

All experiments were performed with a minimum of five independent replicates (n = 5), and data are reported as mean ± standard deviation (mean ± SD). Statistical analyses were carried out and automatically generated using Origin software (OriginPro 2021 (64-bit), version 9.8.0.200).

### 2.9. Characterization of Water Absorption Performance

The 24 h water thickness swelling (TS) was tested according to GB/T 17657-2022 “Test Method for Physical and Chemical Properties of Artificial Boards and Decorative Artificial Boards” [40]. The sample size was 50 mm × 50 mm, with 5 samples tested in each group, and the average value was taken as the result. The change in central thickness of each fiberboard specimen was recorded before and after water immersion. The 24 h water thickness swelling (TS) of the fiberboard, Y (%), was calculated using the following equation:

(2) $$Y=\frac{Z-z}{z}\times 100\%,$$

where Z and z represent the thickness of the fiberboard after and before water immersion, respectively.

### 2.10. Water Contact Angle Test

The water contact angle of the sample was measured using a SPCA-X3 contact angle meter (manufactured by Beijing HARKE Experimental Instrument Factory, Beijing, China).

### 2.11. Detection of Formaldehyde and Total Volatile Organic Compounds (TVOC) Concentrations

Formaldehyde and TVOC concentrations were determined using a 723P spectrophotometer (manufactured by Beijing Guanghua Qimingfeng Technology Co., Ltd., Beijing, China) and a thermal desorption–gas chromatography–mass spectrometry (TD–GCMS) system (GC–MS: QP2010Ultra, manufactured by Shimadzu Corporation, Kyoto, Japan). Measurements were conducted on poplar fiberboard in accordance with the national environmental protection standard HJ 571-2010 ”Technical Requirements for Environmental Labeling Products—Wood-based Panels and Products” [41]. According to the Chinese national standard GB/T 39600-2021 “Classification of Formaldehyde Emission of Wood-Based Panels and Their Products” [42], the formaldehyde emission limit for achieving the ENF (Extra No Formaldehyde) grade is ≤0.025 mg/m^3^.

## 3. Results and Discussion

### 3.1. Microstructural and Chemical Characterization

**Figure 2:**
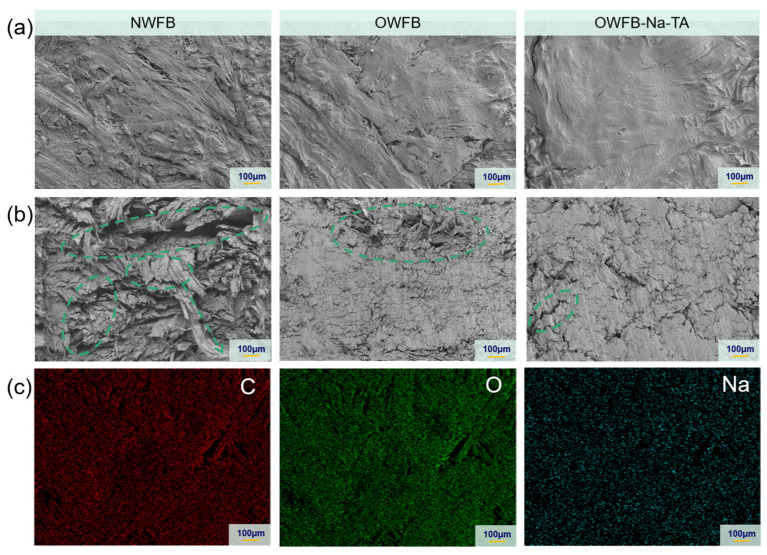
(**a**) Surface SEM images of fiberboards prepared at different fabrication stages (NWFB, OWFB, and OWFB-Na-TA); (**b**) Cross-sectional SEM images of fiberboards prepared at different fabrication stages (The green circles indicate the fibers); (**c**) Energy dispersive X-ray spectroscopy (EDS) elemental mapping images of OWFB-Na-TA.

Figure 2 presents the microstructural evolution of eucalyptus wood fiberboards at different fabrication stages. As shown in Figure 2a, the natural wood fiberboard (NWFB) exhibits a loose microstructure with largely unconnected fibers, resulting in abundant inter-fiber voids. After hemicelluloses removal and oxidative treatment, the coordination between Na^+^ ions and carboxyl groups on the oxidized fiber surface leads to the formation of OWFB-Na, in which fibers partially bridge the inter-fiber gaps, indicating enhanced interfacial interactions. Upon further incorporation of tannic acid (TA), supramolecular networks are constructed through synergistic hydrogen bonding and ionic coordination, and subsequent hot-pressing eliminates the inter-fiber voids, yielding a highly compact and dense microstructure. The corresponding fracture surface SEM images in Figure 2b further confirm the progressive enhancement of inter-fiber bonding. The fracture surface of NWFB shows obvious fiber pull-out and breakage, suggesting weak bonding between fibers. In contrast, the oxidatively treated sample exhibits a relatively smooth fracture surface with indistinct fibrous features. After the introduction of TA and Na^+^ ions, the fracture surface becomes extremely smooth, and individual fibers are no longer observable, indicating the formation of an integrated and cohesive bonding structure. Furthermore, the Energy Dispersive X-ray Spectroscopy (EDS) mapping results in Figure 2c show that, in OWFB-Na-TA, in addition to carbon and oxygen, sodium ions are uniformly distributed throughout the fiberboard matrix. This provides direct evidence for the successful incorporation of Na^+^-mediated supramolecular interactions within the board.

**Figure 3:**
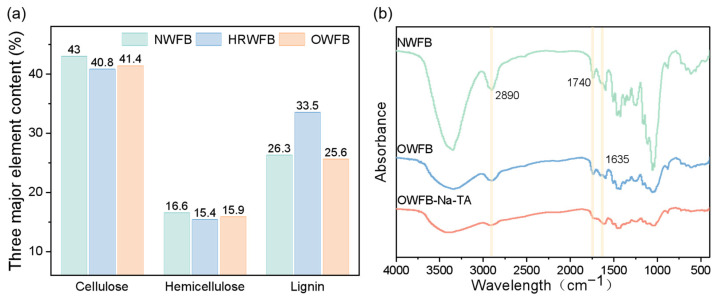
(**a**) Chemical composition analysis showing the relative contents of cellulose, hemicelluloses, and lignin in NWFB, Hemicelluloses-Removed Wood Fiberboard (HRWFB), and OWFB; (**b**) FTIR spectra of NWFB, OWFB, and OWFB-Na-TA.

As shown in Figure 3a, after hemicelluloses removal treatment, the relative content of hemicelluloses decreased from 16.6% to 15.4%, while the relative content of lignin increased from 26.3% to 33.5%. Following oxidative treatment, the relative hemicelluloses content slightly increased from 15.4% to 15.9%, indicating no significant change, whereas the relative lignin content decreased markedly from 33.5% to 25.6%. This decrease in relative lignin content can be attributed to the chemical nature of the oxidation process, which not only converts hydroxyl groups into carboxyl groups on the cell wall polymers but may also partially degrade or solubilize some lignin components, resulting in a lower relative proportion in the solid residue. Minor measurement fluctuations may also contribute to the apparent decrease. Figure 3b presents the Fourier transform infrared (FTIR) spectra of NWFB, OWFB, and OWFB-Na-TA. Compared with NWFB, the characteristic absorption peak at 1740 cm^−1^ in OWFB disappears after chemical treatment, confirming that most hemicelluloses in the cell wall was removed. Meanwhile, a new absorption peak appears at 1635 cm^−1^, which may correspond to the stretching vibration of C=O groups, indicating the possible formation of oxidized functional groups on the fiber surface [37]. Together, the chemical composition analysis and FTIR results demonstrate that the oxidative treatment effectively modifies the fiber cell wall, while partially altering lignin content.

### 3.2. Mechanical and Physical Properties

**Figure 4:**
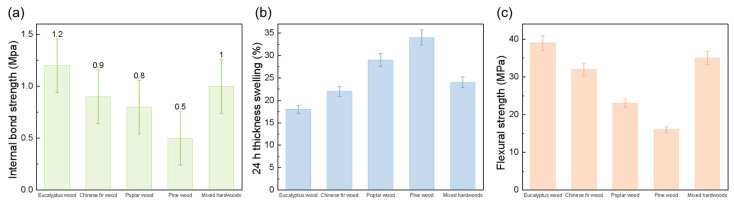
Performance of fiberboards prepared from five different wood species: Eucalyptus, Chinese fir, Poplar, Pine, and Mixed hardwoods. (**a**) Internal bond strength; (**b**) 24 h water thickness swelling; (**c**) Flexural strength.

By comparing the mechanical and physical properties of fiberboards prepared from five different wood species without any chemical treatment, it can be observed that, in Figure 4a, the highest internal bond (IB) strength was obtained for Eucalyptus, followed by mixed hardwoods, while Poplar exhibited the lowest IB. Figure 4b shows the 24 h water thickness swelling, although all samples underwent significant swelling, Eucalyptus and mixed hardwoods swelled less, whereas Poplar reached up to 34%. Combined with the static bending results in Figure 4c, it can be concluded that Eucalyptus and Pine demonstrated relatively good overall performance, whereas Poplar deviated negatively. The inferior performance of Poplar is likely attributed to the inherent differences in fiber size and composition under untreated conditions. Based on these preliminary results, the optimal wood species were selected for further study, while other species could be explored using alternative treatment methods in future work.

**Figure 5:**
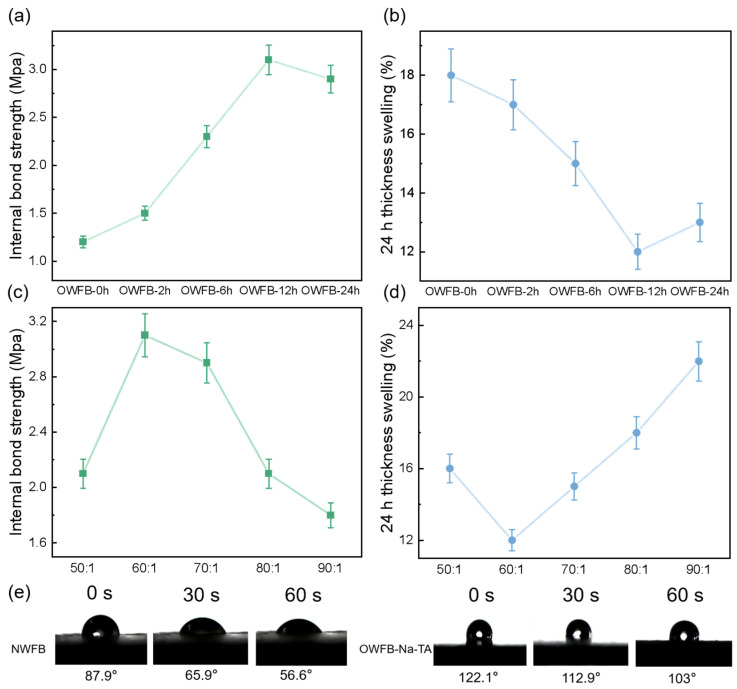
Effects of chemical treatments on fiberboard properties: (**a**) internal bond strength (IB) with different oxidation durations; (**b**) 24 h water thickness swelling with different oxidation durations; (**c**) IB for varying Na^+^ to tannic acid (TA) ratios; (**d**) 24 h water thickness swelling for varying Na^+^/TA ratios; (**e**) hydrophobicity comparison between NWFB and OWFB-Na-TA.

The effects of oxidation time and Na^+^/tannic acid (TA) mass ratio on the mechanical, water-resistance, and hydrophobic properties of fiberboards are presented in Figure 5. As shown in Figure 5a, the internal bond (IB) strength of OWFB increased with oxidation time, reaching a maximum of 3.1 MPa at 12 h, followed by a slight decrease at 24 h, indicating that moderate oxidation enhances fiber–fiber bonding. Correspondingly, the 24 h water thickness swelling (TS) decreased with oxidation time, with the lowest swelling (12%) observed at 12 h (Figure 5b), demonstrating improved water resistance. The effect of varying the Na^+^ to TA mass ratio is illustrated in Figure 5c,d. The IB strength peaked at a ratio of 60:1 (3.1 MPa) and decreased at higher or lower ratios, while the TS exhibited a minimum at the same ratio (12%), indicating that this ratio optimizes both mechanical strength and dimensional stability. The hydrophobicity of the untreated (NWFB) and modified fiberboards (OWFB-Na-TA) is shown in Figure 5e. The contact angle of NWFB decreased from 87.9° to 56.6° over 60 s, whereas OWFB-Na-TA exhibited higher initial contact angles (122.1°) and maintained relatively large values (103°) after 60 s, confirming a significant enhancement in surface hydrophobicity following chemical modification. Overall, these results indicate that 12 h oxidation combined with a 60:1 Na^+^:TA mass ratio provides the optimal balance of internal bond strength, water resistance, and hydrophobicity for the prepared fiberboards.

**Figure 6:**
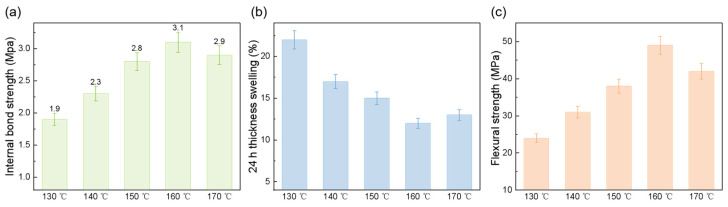
Effects of hot-pressing temperature (130 °C, 140 °C, 150 °C, 160 °C, and 170 °C) on the properties of fiberboards: (**a**) internal bond (IB) strength, (**b**) 24 h water thickness swelling, and (**c**) flexural strength.

Figure 6 illustrates the effects of different hot-pressing temperatures on the mechanical properties and water resistance of fiberboards. As shown in Figure 6a, the internal bond (IB) strength of OWFB increases with rising hot-pressing temperature, reaching a maximum of approximately 3.1 MPa at 160 °C, followed by a slight decrease at 170 °C, indicating that excessively high temperatures may slightly impair fiber–fiber bonding. Correspondingly, the 24 h water thickness swelling (TS) decrease with increasing temperature, with the lowest TS (12%) observed at 160 °C (Figure 6b), demonstrating a significant improvement in water resistance. The effect of hot-pressing temperature on static bending strength is shown in Figure 6c, where the maximum value of 49 MPa is also observed at 160 °C, further confirming that this temperature condition provides optimal mechanical performance. In summary, hot-pressing temperature plays a critical role in regulating fiberboard properties. A moderate temperature of 160 °C achieves the best balance among IB strength, bending strength, and water resistance, whereas temperatures that are too high or too low may compromise mechanical performance or water resistance. These results highlight that precise control of hot-pressing temperature is essential for ensuring the overall performance of fiberboards during fabrication.

### 3.3. Analysis of Odor Characteristics

**Figure 7:**
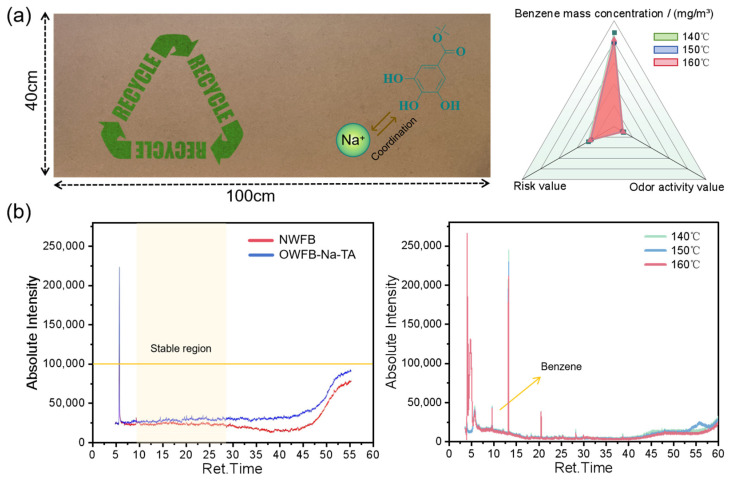
(**a**) Photograph of the prepared fiberboard samples, showing mass concentration, odor activity value (OAV), and risk value; (**b**) VOC composition analysis of NWFB and OWFB-Na-TA; odor analysis at hot-pressing temperatures of 140 °C, 150 °C, and 160 °C.

As shown in Figure 7, all chromatographic peaks of the fiberboard samples before and after oxidative treatment were integrated, and volatile odor compounds were qualitatively identified based on their retention times, retention indices, and similarity values, while solvent-related peaks and siloxane peaks originating from the chromatographic column were excluded [43,44]. As shown in Figure 7a, after screening based on a similarity threshold (similarity ≥ 80), both odor activity values and risk values were very low, and benzene was identified as the only detectable volatile odor compound, with an extremely low concentration. As shown in Figure 7b, the formaldehyde emissions of fiberboards prepared before and after oxidative treatment were 0.005 and 0.006 mg/m^3^, respectively, both below the ENF (Extra No Formaldehyde, formaldehyde emission limit ≤0.025 mg/m^3^) grade. According to the quantitative determination method described in Section 2.10, the total volatile organic compounds (TVOCs) were <0.01 mg/m^3^, with concentrations below the detection limits [45]. In the total ion chromatograms (TIC) of fiberboards prepared at different hot-pressing temperatures (140 °C, 150 °C, and 160 °C), the relatively high-intensity peaks mainly originated from siloxanes in the column, while the characteristic peak of benzene appeared at approximately 9.563 min. These results indicate that boards prepared at different temperatures all exhibit low odor. The reduced odor emission of OWFB–Na–TA fiberboards is attributed to the supramolecular interactions between tannic acid and sodium ions. The phenolic hydroxyl groups of tannic acid enable hydrogen bonding and noncovalent coordination, and the introduction of Na^+^ contributes to the stabilization of the supramolecular network. The reduced odor emission observed for OWFB–Na–TA is associated with the supramolecular interactions between tannic acid and sodium ions. The phenolic hydroxyl groups of tannic acid enable hydrogen bonding and noncovalent coordination, and the introduction of Na^+^ contributes to the stabilization of the supramolecular network. These reversible and dynamic interactions limit the mobility and release of odor-related small molecules, leading to lower VOC intensity and a more stable emission behavior compared with NWFB. This odor reduction arises from molecular-level regulation rather than conventional physical encapsulation or masking.

## 4. Conclusions

This study demonstrates that oxidative pretreatment combined with Na^+^ and TA-mediated supramolecular assembly is an effective strategy to produce high-performance, low-odor, and recyclable high-density fiberboards. The oxidation step introduces active carboxyl groups, which facilitate the formation of robust supramolecular inter-fiber networks through coordination and hydrogen bonding, eliminating the need for formaldehyde-based adhesives. Optimized HDF exhibited high internal bond and bending strengths, minimal thickness swelling, and trace VOC emissions, confirming both superior mechanical performance and environmental safety. Moreover, the reversible Na^+^-TA interactions enable efficient fiber separation and recyclability under mild aqueous conditions. Overall, this work provides a sustainable platform for designing formaldehyde-free wood composites with enhanced functionality, addressing both performance and environmental concerns.

## Data Availability

The original contributions presented in this study are included in the article. Further inquiries can be directed to the corresponding authors.
